# Hot Potatoes in AF

**DOI:** 10.2174/157340312803760776

**Published:** 2012-11

**Authors:** Adrian Baranchuk, Bulent Gorenek, George D Veenhuyzen, Jane Caldwell, Jane Caldwell

**Affiliations:** 1 Department of Cardiology, Kingston General Hospital, Queen's University, Kingston, Ontario, Canada; 2 Department of Cardiology, Faculty of Medicine, Eskisehir Osmangazi University, Eskisehir, Turkey; 3 Libin Cardiovascular Institute of Alberta, University of Calgary, Alberta, Canada

Western society is at the brink of an atrial fibrillation (AF) explosion. Already AF is the most common sustained arrhythmia worldwide; in the US alone the estimated prevalence in 2010 was between 2.7 to 6.1 million, and this is expected to rise to between 5.6 and 12 million by 2050 [1]. The source of this increase is multifactorial and our current ability to manage the condition is often suboptimal. With this in mind we present this dozen of current “hot potatoes” in AF from epidemiology through management to, dare we say it foremost given our current economic climate, the cost implications of our management decisions.

As a society we are becoming more obese and more diabetic, which as discussed by Ashgar *et al.* [2] are both independently linked to AF development. In their review these authors also point out that as well as diabetes, obesity also creates a procoagulant environment as well as posing many practical difficulties in management strategies such as external cardioversion and catheter ablation. They also briefly discuss the link with obesity and obstructive sleep apnoea (OSA), an AF risk factor that is the sole focus of the article by Digby & Baranchuk [3]. OSA is vastly underdiagnosed and is a key factor in the development of AF, recurrence after cardioversion and ablation which can be easily ameliorated in those who are diagnosed and treated with continuous positive airway pressure. In this eloquent article the authors challenge us to look for OSA in all our AF patients as treatment can reverse the atrial remodelling and potentially improve our AF therapies.

Our population is also ageing and suffering from more heart failure, both systolic and diastolic. Khan *et al.* review the complex interplay of heart failure and AF, two modern day epidemics [4]. The authors also review the current controversies in how best to manage AF in this sicker group of people in whom many medications are contraindicated and ablation strategies are associated with increased rates of complications. Adverse events are also key in the interplay of AF with acute coronary syndromes (ACS). Reviewing this topic, Gorenek & Kudaiberdieva point out the association of both pre-existent AF and new AF with unfavourable outcomes in ACS [5]. Whilst AF is associated with worse survival, it is more a reflection of the severity of the ACS event so that therapies should focus on the ACS event rather than on AF.

Modern day management of AF consists of medical management including thromboembolic prophylaxis, invasive catheter ablation and a combination of the two. In both areas there has been a big jump in available drugs and technologies within the last few years. As we are all aware, the search for an oral warfarin replacement is now over, though the reality of day-to-day management and new risk profiles with these replacements is something to think about. Ahmad & Lip excellently review the practicalities of stroke prevention in 2012, both in terms of the newly available anticoagulants but also the new criteria for their usage [6]. In a similar vane, Saklani & Skanes review the wealth of new antiarrhythmic medications that have become available [7]. They outline the highs and lows of the Dronedarone story and look forward to possible future weapons in our AF armamentarium such as Vernakalant and Ranolazine.

Catheter ablation has advanced in the last decade that it is now the therapy of choice for paroxysmal AF that is symptomatic and drug resistant. In this special issue we have 5 papers that reflect the breadth of topical issues in this treatment strategy. First, we have a beautifully illustrated presentation on the anatomy of AF by Sánchez-Quintana *et al*., where they ruminate over the anatomical basis for the triggers and substrate that are essential for AF initiation and maintenance [8]. The shear variation between patients helps us to understand the challenges of AF ablation. Next we have an excellent discourse from our colleagues in Bordeaux who started the whole thing off [9]. Here Roten *et al.* reflect the limitations of AF ablation in both paroxysmal and persistent AF and look at the new techniques, new technologies and strategies that may improve outcomes in the future. One of the strategies they explore is complex fractionated electrogram (CFAE) ablation which is the focus of the article by Caldwell & Redfearn [10]. Heterogeneity of the methods to detect CFAE, ablation strategies and approaches to differentiate functional from bystander fractionated signals are all hot potatoes that current persistent AF ablators have to handle, as is the issue of how to define the long term success of their efforts. Kircher *et al.* discuss the dilemma that the longer we wait and longer periods we look over the more we find AF recurrence even if the patient is asymptomatic [11]. Yet despite this we are attempting ablation in patients who were previously thought to be unablatable. Santangeli *et al.* discuss their own and the worldwide experience of performing AF ablation in patients with metallic mitral valve replacements, a thought that still puts shivers down many ablators’ spines [12]. Their success and lack of complications are remarkable.

The final “hot potato” by Khaykin & Shamiss [13] brings us full circle by examining the cost implications of AF. This detailed report compares the cost and consequences of invasive vs non-invasive management of AF concluding that “While more expensive up front, ablation appears to be a cost-effective alternative to the non-invasive AF treatment strategies after a 3-5 year time horizon”. The question is whether governments are willing to take a more long term view on managing AF or whether the current financial constraints will force them to take less costly temporising approach to AF management.
